# A themed collection in memory of Petr Nachtigall

**DOI:** 10.1039/d3ra90017g

**Published:** 2023-03-17

**Authors:** Lukáš Grajciar, Christopher J. Heard, Russel E. Morris, Joachim Sauer, Jiří Čejka

**Affiliations:** a Department of Physical and Macromolecular Chemistry, Faculty of Science, Charles University Hlavova 8, 128 43 Praha 2 Czech Republic; b University of St Andrews, School of Chemistry Purdie Building, St Andrews Fife KY16 9ST Scotland; c Humboldt University, Inst. Chem. D-10099 Berlin Germany

## Abstract

Professor RNDr. Petr Nachtigall, PhD passed away on 28 December 2022. He was an internationally recognized expert in computational materials science; working at Charles University in the Department of Physical and Macromolecular Chemistry. We honor his memory.
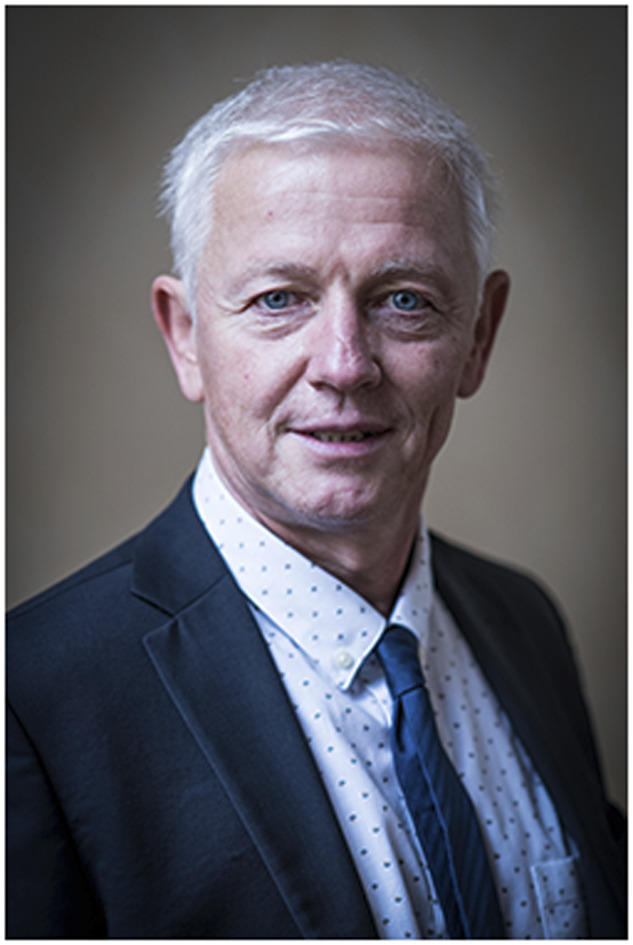

Professor Petr Nachtigall was an internationally recognized expert in computational physical and materials science. He received his PhD at the University of Pittsburgh. After returning to Prague, he worked at the J. Heyrovsky Institute of Physical Chemistry, later at the Institute of Organic Chemistry and Biochemistry of the Czech Academy of Sciences, and since 2009 at the Faculty of Science of Charles University where he was a major leader in the Charles University Centre for Advanced Materials. Petr has regularly published in the most prestigious scientific journals: he is the author of 181 well-recognized publications.

Petr was a computational chemist who liked to solve materials chemistry problems in close collaboration with experimentalists and he was very successful in that. Where necessary, he implemented techniques beyond routine methodology. In particular, he found ways to overcome the limitations of density functional theory. Petr had wide-ranging expertise in computational chemistry and he advanced the understanding of adsorption complexes and reaction mechanisms in zeolites and metal–organic frameworks at the molecular scale. His studies of new types of two-dimensional materials lead to an improved understanding of their very interesting properties, for example the superior lithium-storage properties of silicene nanosheets or utilization of two-dimensional zeolites for fabrication of new materials.

Petr’s theoretical insight into the synthesis of new materials contributed significantly to the discovery and theoretical explanation of the new ADOR (assembly-disassembly-organisation-reassembly) method of zeolite synthesis, thanks to which more than 10 new types of zeolites have already been synthesized. Petr received several awards during his career, including the Bedřich Hrozný Prize for Basic Research (2015), the Prize of the Czech Minister of Education, Youth and Sports for significant results of basic research in natural sciences (2019) and the Donatio Universitatis Carolinae (2021).

In Petr Nachtigall we lose not only an outstanding scientist, teacher, and mentor, but also a great person, a dear colleague and friend.

## Supplementary Material

